# Knockout of Nur77 Leads to Amino Acid, Lipid, and Glucose Metabolism Disorders in Zebrafish

**DOI:** 10.3389/fendo.2022.864631

**Published:** 2022-04-25

**Authors:** Yang Xu, Juanjuan Tian, Qi Kang, Hang Yuan, Chengdong Liu, Zhehui Li, Jie Liu, Mingyu Li

**Affiliations:** ^1^ Fujian Provincial Key Laboratory of Innovative Drug Target Research, School of Pharmaceutical Sciences, Xiamen University, Xiamen, China; ^2^ State Key Laboratory of Cellular Stress Biology, School of Life Sciences, Xiamen University, Xiamen, China; ^3^ Key Laboratory of Mariculture (Ministry of Education), Ocean University of China, Qingdao, China

**Keywords:** Nur77, zebrafish, transcriptomics, β cells, lipid metabolism, glucose metabolism

## Abstract

Orphan nuclear receptor Nur77 has been reported to be implicated in a diverse range of metabolic processes, including carbohydrate metabolism and lipid metabolism. However, the detailed mechanism of Nur77 in the regulation of metabolic pathway still needs to be further investigated. In this study, we created a global *nur77* knockout zebrafish model by CRISPR/Cas9 technique, and then performed whole-organism RNA sequencing analysis in wildtype and *nur77*-deficient zebrafish to dissect the genetic changes in metabolic-related pathways. We found that many genes involved in amino acid, lipid, and carbohydrate metabolism changed by more than twofold. Furthermore, we revealed that *nur77^−/−^* mutant displayed increased total cholesterol (TC) and triglyceride (TG), alteration in total amino acids, as well as elevated glucose. We also demonstrated that the elevated glucose was not due to the change of glucose uptake but was likely caused by the disorder of glycolysis/gluconeogenesis and the impaired β-cell function, including downregulated *insb* expression, reduced β-cell mass, and suppressed insulin secretion. Importantly, we also verified that targeted expression of Nur77 in the β cells is sufficient to rescue the β-cell defects in global *nur77^−/−^* larvae zebrafish. These results provide new information about the global metabolic network that Nur77 signaling regulates, as well as the role of Nur77 in β-cell function.

## Introduction

Nuclear receptor Nur77, also known as TR3, NR4A1, NGFI-B, NAK1, or TIS1 belongs to the NR4A subfamily of the nuclear receptor superfamily (1, 2). Nur77 is an early immediate response gene which is induced by a diverse range of physiological molecules and stimuli, including cytokines, growth factors, neurotransmitters, stress, glucose, and fatty acids (3–6). It plays a pleiotropic role in cell proliferation, differentiation, inflammation, immunity, apoptosis, and metabolism (2, 7, 8).

Recently, more and more investigations have characterized Nur77 as a key regulator to control glucose and lipid homeostasis. Nur77 is expressed in a wide variety of metabolically related tissues, such as skeletal muscle, liver, pancreas, and adipose (9–15). In skeletal muscle cells, diminished Nur77 expression restrains lipolysis and lipid, carbohydrate, energy metabolism-associated genes and protein expression (16). Overexpression of Nur77 results in the predominant augmented levels of glucose transport, insulin signaling (such as Glut4), glycolysis, and glycogenolysis-related genes (17–20). Nur77 overexpression can enhance glucose transport (21) and alter muscle mass and myofiber size through regulation of glycolysis in mice (22). Nur77 null mice with high-fat feeding exhibit metabolic changes like decreased energy usage, insulin resistance, lower blood glucose clearance, and increased lipid content in skeletal muscle (23).

Not only in skeletal muscle, but Nur77 also plays a similar metabolic role in the liver (18). High expression of Nur77 drives hepatic glucose production, raises blood glucose levels, and induces genes involved in gluconeogenesis (18, 24). Nur77 null mice with high-fat feeding also exhibits decreased hepatic glucose production and liver insulin resistance (23). Nur77 agonists, such as cytosporone B (CsnB), could raise blood glucose in mice (25). Nur77 is also involved in the modulation of lipid metabolism in the liver. In a gain-of-function approach, hepatic overexpression of Nur77 decreases hepatic triglyceride by suppressing sterol regulatory element-binding protein 1c (SREBP1c) and altering other lipogenic enzyme gene expression (26). Nur77 null mice with high-fat feeding exhibit upregulated hepatic steatosis and high expression levels of lipogenic genes in the liver (18).

In adipose tissue, the expression level of Nur77 is significantly lower in the adipocytes of obese and diabetic mice (27). Nur77 also exhibits a negative regulation to adipocyte differentiation, lipid accumulation, and adipogenesis in adipocyte progenitor (28–30). Nur77-deficient mice exhibit increased susceptibility to high-fat diet (HFD)-induced obesity, thus higher body weight and fat mass are observed. What really matter is celastrol significantly reduced body weight, adipose tissue mass, and the size of adipocytes caused by HFD that was suppressed in Nur77-deficient mice.

Most recently, the important role of Nur77 in pancreatic β cells has been examined. Nur77 protects pancreatic β cells by attenuating endoplasmic reticulum (ER) stress and oxidative stress-induced apoptosis (31–34). In contrast, glucose and palmitate induce Nur77 expression in pancreatic β-cell lines, Ins-1 and MIN6 (35–37). Increased Nur77 expression level decreases intracellular insulin content, and glucose-stimulated insulin secretion is detrimental to β cell (37).

Therefore, Nur77 is important in the regulation of lipid and carbohydrate metabolism in the key metabolic tissues. Some small molecules, such as CsnB (25) and celastrol (32), have been improved to regulate metabolism in Nur77-dependent pathway. These studies demonstrate that new Nur77-targeted drugs may have great potential in therapeutic utility against metabolic disease, obesity, dyslipidemia, and cardiovascular disease.

Appropriate screening models are essential for drug development. Zebrafish has high genetic homology and similar development of several organs to humans. It is convenient to get sufficient embryos and larvae for global systematic studies and drug screening. Although the omics studies have been applied in *Nur77^−/−^* mice (23, 38), the whole animal transcriptome, especially the metabolic alterations are still unclear. In the current investigation, we first generated *nur77* knockout zebrafish and applied RNA sequencing (RNA-Seq) analysis using the whole zebrafish larvae. The transcriptome results could give us a global view of the alteration of gene expression in the absence of *nur77*. We aimed to illuminate comprehensive metabolic alterations in *nur77^−/−^* zebrafish. Also, *nur77* knockout mutant line should facilitate a further mechanism study and drug screening of Nur77-associated metabolic regulation.

## Materials and Methods

### Zebrafish Lines and Maintenance

Zebrafish (*Danio rerio*) were cultured in a circulating water culture system (Shanghai Haisheng Biotechnology Co. Ltd., Shanghai, China) with a water temperature of 28°C and raised in a 14-h light and 10-h dark cycle. The embryos were obtained from natural culture and reared in 28.5°C embryo culture medium according to the method of Kimmel et al. (39). In this study, AB strain, *nur77* mutant zebrafish, *Tg(gcga:GFP)* (40), *Tg(Ins:H2BmCherry)* (41), and (*Tg Ins:GCaMP6s: eGFP*) (42) transgenic lines were used as the research object. All procedures have been approved by the Animal Care and Utilization Committee of Xiamen University (Protocol No. XMULAC20160089 of March 10, 2016).

### Establishment of *nur77* Knockout Zebrafish Using CRISPR/Cas9 Technique

CRISPR/Cas9 technology was applied to edit the genome of zebrafish embryos. The sgRNA target site (CCGGGCAGCTGGACTCCTTC) was designed by using the online tool CRISPRscan. *In vitro*, sgRNA was synthesized from T7 kit (MAXIscript T7 Transcription Kit, Invitrogen, Carlsbad, CA, USA) and purified by RNA purification kit (RNA Clean and Concentrator-5, Zymo Research, Irvine, CA, USA). sgRNA and Cas9 protein (NEB, Beijing, China) were then coinjected into single-cell stage embryos. Genomic DNA was extracted from the embryos to evaluate the efficiency of genome editing. The genomic region around the target site of CRISPR was amplified by polymerase chain reaction (PCR), and the mutation was detected by electrophoretic gel image (5′ to 3′end of primer sequence is F: CTCCCTCTTCAGCTCAGAGTTTCT, R: TGCAGATGCCGGACTTCCA). The mutant F0 generation was raised to sexual maturity, and the F1 generation zebrafish was obtained by mating with AB zebrafish. The genomic DNA of F1 generation was extracted and the genomic region around the CRISPR target site was amplified by PCR, and the single mutant zebrafish with 13 bp gene (mutant sequence ACCTCCGGGCAGC) was obtained by sequencing. The zebrafish strain of *nur77* mutant was obtained by further hybridization.

### RNA Extraction, cDNA Library Preparation, and Sequencing

TRIzol reagent (Thermo Fisher Scientific, Waltham, MA, USA) was used to extract total RNA from 40 zebrafish larvae and repeated three times. In order to remove any genomic DNA contamination, RQ1 RNase-Free DNase (Promega, Madison, WI, USA) was applied to purify RNA. The concentration and mass of each sample were determined by Agilent RNA6000 nano-kit in Agilent 2100 Biological Analyzer (Agilent Technologies, Santa Clara, CA, USA). The RNA mass fraction (RIN/RQN) of all samples is 10.

The mRNA of each sample was selected by oligomeric (dT) beads to construct the cDNA libraries. The total mRNA fragments were inversely transcribed into double-stranded cDNA (Ds cDNA) with N6 random primers. The synthesized double-stranded cDNA underwent terminal repair, 5′-terminal phosphorylation, and 3′-adenylation. The splice is then connected to the 3′-terminal adenylate cDNA fragment. The ligated products were used for PCR amplification to enrich the cDNA templates. After denaturation of these PCR products, the single-stranded DNA was cyclized to form the final cDNA library. All cDNA libraries were sequenced on the BGISEQ-500RS platform (BGI, Shenzhen, China) in accordance with the manufacturer’s standard agreement.

### Bioinformatics Analysis of RNA Sequence Data

The original data were tested by BGISEQ-500 quality control and filtered into clean readings using SOAPnuke software. This process discards readout containing adaptation sequences, readout containing unknown base “N” more than 10%, and low-quality readout (the percentage of low-quality bases in readout is more than 50%). After filtering, the clean readings are compared with the zebrafish genome (*Danio rerio*, GRzh11, http://asia.ensembl.org/Danio_rerio/Info/Index), using HISat (v2.0.4). After comparison, Bowtie2 and RSEM (RNA-Seq by Expect Maximization) for clean reading of zebrafish single genes were used to quantify gene expression by using the fragments per kilobase per million (FPKM) method (fragments per kilobase transcript/million sequenced fragments). In addition, some unidentified genes were annotated in Ensembl (www.ensembl.org) by BLAST and Synteny analysis. These sequence data are deposited in the NCBI database and can be obtained through BioProject ID : PRJNA801348.

### Analysis of Differentially Expressed Genes

DESEQ2 is used to identify differentially expressed genes (DEGs). The genes with fold change ≥2.00 and adjusted *p*-value ≤0.05 were regarded as DEG with statistical significance. For repeated samples, the log2 multiple change (Log2FC) and probability of each gene in each comparison were calculated by NOISEQ under the condition of fold change ≥2.00 and probability ≥0.80.

Gene Ontology (GO) and Kyoto Encyclopedia of Gene and Genome (KEGG) databases were used for pathway enrichment analysis. The significantly enriched genes are those with *q*-value ≤0.001 and FDR ≤0.01.

### Quantitative RT-PCR

DEGs are verified by quantitative RT-PCR (qRT-PCR) technology. The extracted RNA was reverse transcribed with oligonucleotide primer (dT) 16 and M-MLV (Promega), and the first-strand cDNA was synthesized. The qRT-PCR reaction is carried out in the Agilent AriaMx system (Agilent Technologies) using PowerUp SYBR Green Master Mix (Thermo Fisher Science). The amplification procedure was 95°C for 10 s and 60°C for 30 s and a total of 40 cycles. Three groups of wildtype or *nur77^−/−^* samples were used, and each sample was tested in three parallel copies. In this experiment, the mRNA level was calculated by 2^−ΔΔ^Ct method, and the level of each gene relative to the internal reference (fold) was calculated by using the β-actin level as an internal reference. These data are provided by the average of three biological samples in each group. The primers used in qRT-PCR are listed in **Supplementary Table S1**.

### 2-[N-(7-Nitrobenz-2-oxa-1,3-Diazol-4-yl) Amino]-2-Deoxy-d-Glucose (2-NBDG) Uptake Test

Zebrafish larvae around 6 days postfertilization (dpf) were incubated in 0.3× Danieau solution containing 600 μM 2-NBDG (B6035, Apexbio, Houston, Texas, USA) for 3 h and then anesthetized and immediately imaged under M205FCA microscope (Leica, Wetzlar, Germany). Finally, the fluorescence intensity of zebrafish eye lens was used to indicate the glucose uptake according to Lee et al. (43).

### Determination of Free Glucose

Free glucose was determined by a glucose detection kit (Red Glucose/Glucose Oxidase Assay Kit, Invitrogen, Carlsbad, CA, USA). Ten larvae were homogenized in 100 μl sample buffer, and the supernatant was absorbed after centrifugation. The free glucose equivalent to one larva (10 μl homogenate) was determined according to the manufacturer’s instructions. At least there were 3 independent biological repeats for each sample.

### Determination of Total Cholesterol and Total Triglycerides

The total cholesterol test kit (A111-1, Nanjing Jiancheng Bioengineering Research Institute, Nanjing, China) and triglyceride test kit (A110-1-1, Nanjing Jiancheng Bioengineering Research Institute) were used to determine the total cholesterol in zebrafish. Thirty 6 dpf zebrafish larvae were randomly selected from wildtype (WT) and *nur77^−/−^* groups and collected. Each group had 3 independent biological repeats. The total cholesterol and total triglyceride were measured according to the instruction of the kit.

### Amino Acid Measurement

Seventy embryos of 6 dpf from WT and *nur77^−/−^* groups were randomly harvested, with three independent biological repeats in each group. The sample was freeze-dried and hydrolyzed in 6 N HCl at 110°C for 24 h. The distilled water was then added to adjust the volume to 10 ml. One-milliliter hydrolysate was dried by nitrogen blower and suspended in 1 ml 0.02 M HCl. After resuspension, the sample was filtered by 0.22 μm filter membrane, and the amino acid composition was determined by the Japanese Chiyoda Hitachi L8900 amino acid automatic analyzer (Chiyoda Hitachi, Tokyo, Japan). The amino acids in larvae were expressed by micrograms per milligram wet tissue.

### GCaMP6s Image Acquisition and Analysis

GCaMP6s measurements were performed on isolated islet from *Tg(Ins:H2BmCherry); Tg(Ins:GCaMP6s)* and *nur77^−/−^; Tg(Ins:H2BmCherry); Tg(Ins:GCaMP6s)* transgenic animals at 6 dpf. The isolated islets were embedded in agarose gel and immersed in extracellular solution (ECS) containing 5 and 20 mM glucose, respectively. The glucose-stimulated Ca^2+^ influx was imaged by Leica SP8 and analyzed by Image J.

### Counting of β Cells

After fixation in 4% paraformaldehyde overnight at 4°C, larvae were washed with 1× PBS plus 0.1% Tween-20 (PBST) and flat mounted in Aqua-Mount (Richard-Allan Scientific, Kalamazoo, MI, USA) with their right side facing the coverslip. The larvae were flattened just to disrupt the islet slightly to allow better resolution of β cells. The β cells were counted according to the mCherry signal using a Zeiss AxioImager under a ×40 lens or using confocal projections taken by Zeiss LSM710 under a ×40 lens (Carl Zeiss, Oberkohen, baden-Wurberg, Germany).

### Insulin Immunofluorescence Staining

Larvae around 6 dpf were fixed in 4% paraformaldehyde overnight at 4°C. After being rinsed, dehydrated, rehydrated, and acetone processed, the larvae were incubated in block solution (5% FBS in PBDT, 1× PBS, 0.1% Tween-20, 1% DMSO) for 2 h at RT. The primary antibody used in this study was anti-insulin (guinea pig, Dako A0564, 1:1,000 in 2% FBS PBDT) overnight (O/N) at 4°C. After washing 4× for 10 min in PBDT, larvae were incubated with secondary antibody (goat anti-guinea pig, Invitrogen A11075, 1:1,000 in 2% FBS PBDT) for 2 h at RT. The larvae were flat mounted in Aqua-Mount and imaged using Zeiss LSM710.

### Establishment of Transgenic Line

The *Tg(Ins : Nur77; cmlc2:eGFP)* transgenic line was generated using the miniTol2-based transposon system (44). The transgene was assembled using a multisite Gateway system (45). The human Nur77 cDNA was amplified and subcloned into pME-MCS vector to generate pME-Nur77. The p5E-Ins, pME-Nur77, and p3E-MCS were then assembled in pDestTol2-CG2 destination vector to obtain the final transgene of *Tg(ins:Nur77;cmlc2:eGFP)*. The cmlc2:EGFP element was used to facilitate identification of transgenic carriers. The plasmid of *Tg(ins:Nur77;cmlc2:eGFP)* is mixed with Tol2 transposase mRNA (25 ng/μl each) and injected into zebrafish embryos at the one-cell stage using a standard protocol as described (45). F1 progeny were screened for eGFP expression in the heart and confirmed by PCR using primers Ins-Nur77-F:5’-CCACCACCATATCCACCATT-3’ and Ins-Nur77-R:5’-TCTTCTCCGCCCACTTGC-3’.

### Statistical Analysis

All the raw data were analyzed with GraphPad prism8 software (GraphPad Software, La Jolla, CA, USA). Results are presented as mean values ± SEM. No outlying values were excluded from the datasets used for statistical analysis. The statistics were performed using one-way ANOVA followed by Bonferroni *post-hoc* test or *t*-test (SPSS). *p* < 0.05 was considered significant.

## Results

### Knockout of Nur77 in Zebrafish by CRISPR/Cas9

**Figure 1:**
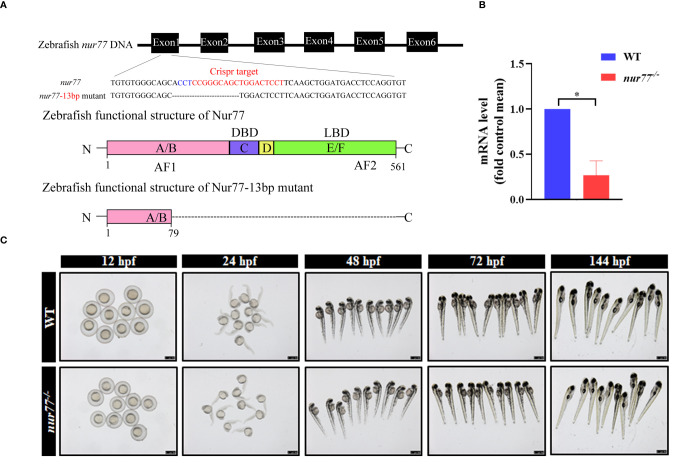
Knockout of *nur77* in zebrafish by Crispr/Cas9. **(A)** Schematic representations of Crispr-Cas9 targets and mutant alleles. *nur77* consists of 6 exons (filled box). The sgRNA target exon 1, and the sequences of the target region were aligned to the selected allele. The target site was in red font, and the PAM site was in blue font. The obtained *nur77^−/−^* zebrafish mutant lacked 13 bp bases, causing protein translation to stop prematurely. **(B)** The validation of the expression level of *nur77* by qRT-PCR analysis (*n* = 3); ^*^ *p* < 0.05 by *t*-test. **(C)** The morphology of WT and *nur77^−/−^* in developmental stages of 12, 24, 48, 72, and 144 hpf. Scale bars indicate 750 μm.

In the zebrafish genome, there is only one ortholog gene of human *Nur77*, which we named *nur77*. To elucidate the function of Nur77 in zebrafish, we then generated *nur77* mutation zebrafish by CRISPR/Cas9 technique. We got a stable mutant line, which had 13 bp deletion in the exon1 of *nur77* (**Figure 1A**). The mutation resulted in reading frame shift and premature stop codon, which disrupted all known functional domains of the Nur77 protein (**Figure 1A**; **Supplementary Figure S1**). qRT-PCR analysis showed that *nur77* mRNA level was dramatically decreased in *nur77^−/−^* zebrafish (**Figure 1B**). The reduction of the mutant mRNA levels is likely due to nonsense-mediated decay of mRNA. We then evaluated the morphological changes in *nur77^−/−^* zebrafish during the different development stages. However, there are no obvious morphological phenotype differences between wildtype and *nur77^−/−^* zebrafish at the stages of 12, 24, 48, 72, and 144 hpf (**Figure 1C**). Moreover, the adult *nur77^−/−^* was morphologically normal and fertilizable (data not shown).

### The Transcriptome of Wildtype and *nur77^−/−^* Larvae

**Figure 2:**
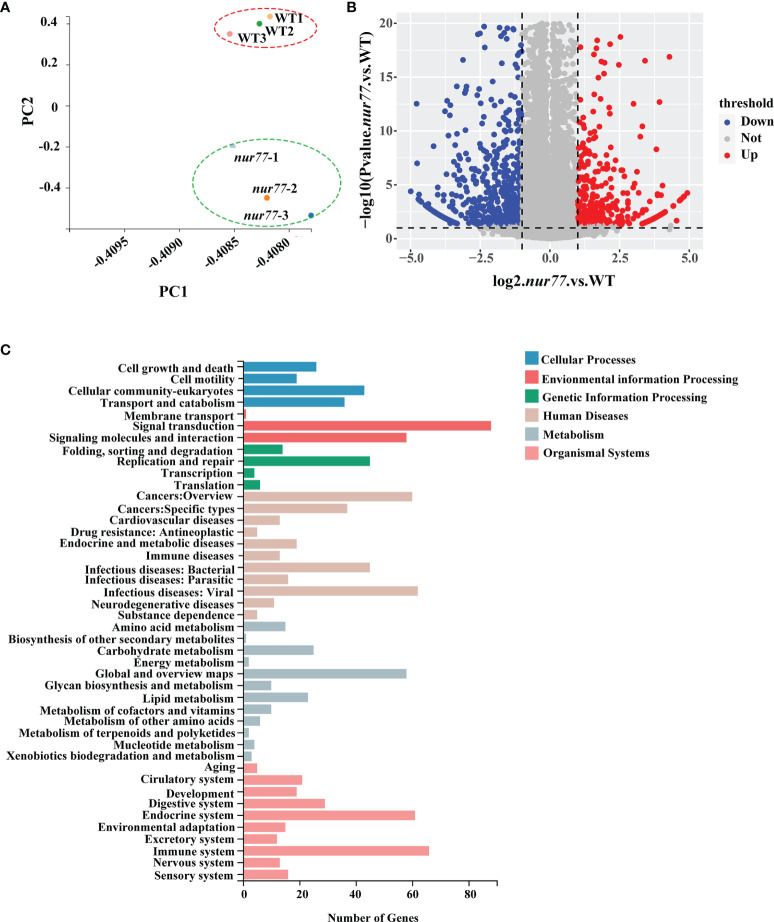
RNA-seq (RNA sequencing) analysis of *nur77^−/−^* mutant zebrafish. **(A)** Principal component analysis (PCA) plot of three wild-type and three *nur77* ^−/−^ mutant RNA-seq datasets. Principal component 1 (PC1) and principal component 2 (PC2) were used for analysis. **(B)** Volcano plot of differential gene expression analysis of *nur77* ^−/−^ mutant and control larvae showing the relationship between *p*-value and log fold changes. Red shows upregulated genes and blue downregulated genes. **(C)** Kyoto Encyclopedia of Genes and Genomes (KEGG) enrichment analysis of DEGs in pathways. The *y*-axis indicates pathways, and the *x*-axis indicates the number of DEGs.

To further explore the function of Nur77 in zebrafish, the total RNAs from 6 dpf wildtype and *nur77^−/−^* larvae were subjected to high-throughput RNA sequencing (RNA-seq). Together, these samples generated 21.52 million (M) pairs of total raw reads and total 25,9235 genes were detected. The average gene mapping ratio was 73.3% (72.2%–74.5%), with genes uniquely mapped between 64.4% and 66.0%. Principal component analysis (PCA) demonstrated that the *nur77^−/−^* mutant datasets clustered distinctly compared with wildtype control (**Figure 2A**). A fold change threshold of 2 while adjusted *q*-value ≤0.001 was used to define DEGs. Comparison of the two genotypes found 580 DEGs, with 190 upregulated and 390 downregulated in *nur77^−/−^* (**Figure 2B**; **Supplementary Table S2**). All the DEGs were annotated with GO terms and assigned into three major functional categories: biological process (BPs), cellular components (CCs), and molecular functions (MFs). “Cellular process” (20%), “metabolic process” (14%), and “biological regulation” (11%) were major items involved in BPs (**Supplementary Figure 2A**). “Cell” (27%), “membrane” (20%), and “organelle” (10%) were top 3 classes in CCs (**Supplementary Figure 2B**). “Binding” (52%) and “catalytic activity” (26%) were the main subcategories in MFs (**Supplementary Figure 2C**). To well understand the GO-annotated DEGs in the *nur77^−/−^* larvae, the 580 DEGs were subjected to KEGG database for canonical signaling pathway analysis. As shown in **Figure 2C**, these DEGs were significantly enriched in 44 different signaling pathways, and these pathways were most related to metabolism (12), human disease (11), organismal systems (10), cellular processes (4), genetic information processing (4), and environmental information processing (3). Since metabolism-related pathways were the most enriched, and *Nur77* is known to be expressed in a wide variety of metabolic-related and energy-demanded tissues and organelles, including the liver, skeletal muscle, adipose, heart, and brain (9–15, 46). We then further analyzed these pathways below.

### Knockout of Nur77 Impaired Amino Acid Metabolism in Zebrafish

**Figure 3:**
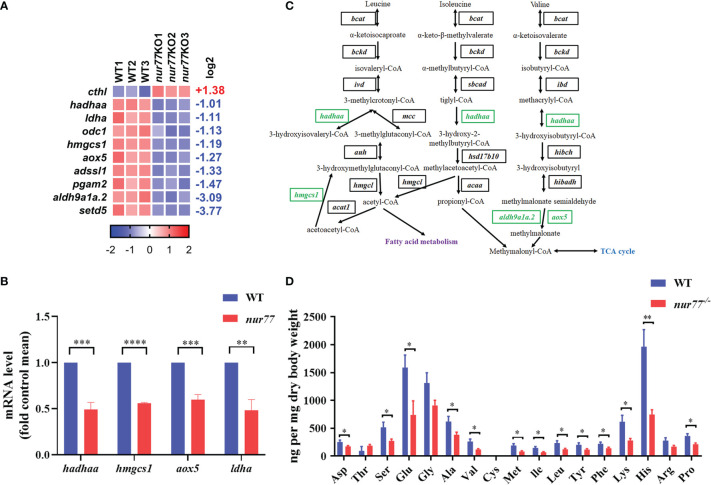
Nur77 regulates amino acid metabolism in zebrafish larvae. **(A)** Heatmaps of transcripts in amino acid metabolism enrichment. Colors represent high (red), low (blue), or average (white) expression values based on Z-score-normalized fragments per kilobase per million mapped read (FPKM) values for each gene. The Z-score indicators are shown under each map. The fold change (log2) is shown on the right. **(B)** The validation of the expression levels of differentially expressed genes by qRT-PCR analysis in the categories of amino acid metabolism (*n* = 3). **(C)** Disturbed valine, leucine, and isoleucine degradation. Blocks represent transcript-encoded enzymes. Green blocks represent downregulated genes, and black blocks represent unchanged genes. **(D)** Amino acids compositions in WT and *nur77* ^−/−^ embryos. Results are represented as means with standard errors (*n* = 3); ^*^ *p* < 0.05; ^**^ *p* < 0.01; ***p < 0.001, ****p < 0.0001 by t-test.

For amino acid metabolism, 10 DEGs were identified in 10 amino acid metabolism pathways (**Figure 3A**). To further evaluate the RNA-seq data, qRT-PCR was applied to detect the selected genes related to amino acid metabolism (*hadhaa*, *hmgcs1*, *aox5*, and *ldha*). Our qRT-PCR results were consistent with the RNA-seq data (**Figure 3B**). Among these 10 DEGs, 4 of them are involved in valine, leucine, and isoleucine degradation (ko00280) (**Figure 3C**; **Supplementary Table S3**), including hydroxyacyl-CoA dehydrogenase trifunctional multienzyme complex subunit alpha a (*hadhaa*), hydroxymethylglutaryl-CoA synthase 1 (*hmgcs1*), aldehyde dehydrogenase family 9 member A1 (*aldh9a1a.2*), and aldehyde oxidase (*aox5*). *hadhaa* is a bifunctional subunit of mitochondrial trifunctional protein and is responsible for the mitochondrial β-oxidation of long-chain fatty acids (47). *hmgcs1* catalyzes the synthesis of HMG-CoA (48). The above genes are involved in the valine, leucine, and isoleucine degradation pathway and contributed to the glucogenic and ketogenic process of these amino acids (49, 50).

As we know, there are almost no reports on the relationship between Nur77 and amino acid metabolism. To further detect the role of Nur77 in amino acid metabolism, the total amino acid compositions of the whole larvae were measured. As shown in **Figure 3D**, valine (Val), leucine (Leu), and isoleucine (Ile) were all decreased. The lower level of these three amino acids may associate with the decreased expression level of genes in valine, leucine, and isoleucine degradation pathway (**Figure 3C**). Moreover, many other amino acids we measured were decreased, except tyrosine (Thr), cysteine (Cys), and arginine (Arg) (**Figure 3D**). All these data suggested the dysregulated amino acid metabolism in *nur77^−/−^* larvae.

### Knockout of Nur77 Impaired Lipid Metabolism in Zebrafish

**Figure 4:**
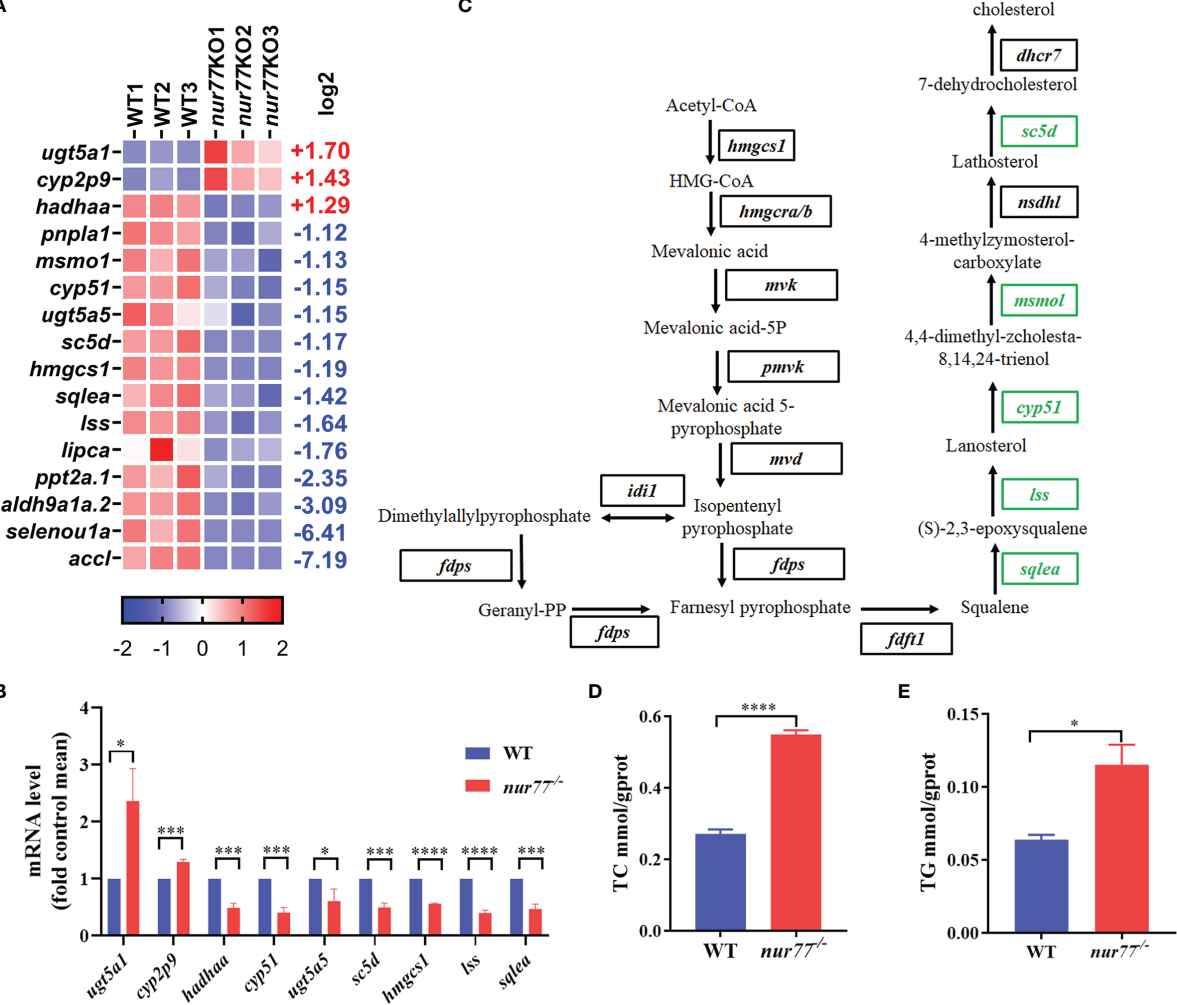
Nur77 regulates lipid metabolism in zebrafish larvae. **(A)** Heatmaps of transcripts in lipid metabolism enrichment. Colors represent high (red), low (blue), or average (white) expression values based on Z-score-normalized fragments per kilobase per million mapped read (FPKM) values for each gene. The Z-score indicators are shown under each map. The fold change (log2) is shown on the right. **(B)** The validation of the expression levels of differentially expressed genes by qRT-PCR analysis in the categories of lipid metabolism (*n* = 3). **(C)** Disturbed cholesterol biosynthesis. Blocks represent transcript-encoded enzymes. Green blocks represent downregulated genes, and black blocks represent unchanged genes. **(D)** The whole-body cholesterol (TC) contents of WT and *nur77* ^−/−^ zebrafish (*n* = 3). **(E)** The whole-body total triglyceride (TG) contents of WT and *nur77* ^−/−^ zebrafish. Results are represented as means with standard errors (*n* = 3); ^*^ *p* < 0.05, ^***^ *p* < 0.001, ^****^ *p* < 0.0001 by *t*-test.

16 DEGs in 11 lipid metabolic pathways were enriched (**Supplementary Table S4**; **Figure 4A**). 5 of them were involved in steroid biosynthesis (ko00100), and 4 of them were involved in glycerolipid metabolism (ko00561) pathways (**Supplementary Table S4**). qRT-PCR analysis was consistence with selected genes in lipid metabolism (*ugt5a1*, *cyp2p9*, *hadhaa*, *cyp51*, *sc5d*, *hmgcs1*, *lss*, *lipca*, and *sqlea*) (**Figure 4B**). Interestingly, all the DGEs involved in the cholesterol biosynthesis pathway were downregulated (**Figure 4C**). Squalene epoxidase a (*sqlea*) catalyzes the first oxygenation step in cholesterol biosynthesis and is thought to be one of the rate-limiting enzymes in this pathway (51). Methylsterol monooxygenase 1 (*msmo1*) plays a role in cholesterol biosynthesis in the liver (52). *cyp51* (sterol 14α-demethylase), as a cytochrome P450, is crucial for biosynthesis reaction of sterols and serves as a drug target in the clinic (53). Lanosterol synthase (*lss*) catalyzes the conversion of (S)-2,3 oxidosqualene to lanosterol and is an essential rate-limiting enzyme in the biosynthesis of cholesterol, steroid hormones, and vitamin D (54, 55). Sterol-C5-desaturase (*sc5d*) converts lathosterol to 7-dehydrocholesterol in cholesterol biosynthesis, ubiquitously expressed in the liver (56). All alteration expression of these genes reflects the reduced cholesterol anabolism.

Although it has been reported that Nur77 null mice fed with high-fat diet were more prone to obesity and Nur77 had important role in the regulation of lipid metabolism in the liver and muscles (23, 26, 29), the whole-body changes in the lipid content are unclear. Here, we evaluated the TC and TG content between the wildtype and *nur77^−/−^* larvae. The significantly elevated TC and TG in nur77*^−/−^* larvae are shown in **Figures 4D, E**. The higher level of cholesterol in *nur77^−/−^* may suppress the cholesterol anabolism, which conversely can decrease the expression of genes in cholesterol biosynthesis pathway (**Figure 4D**). Taken together, these data suggested that Nur77 knockout impaired the lipid metabolism of zebrafish.

### Knockout of Nur77 Impaired Carbohydrate Metabolism in Zebrafish

**Figure 5:**
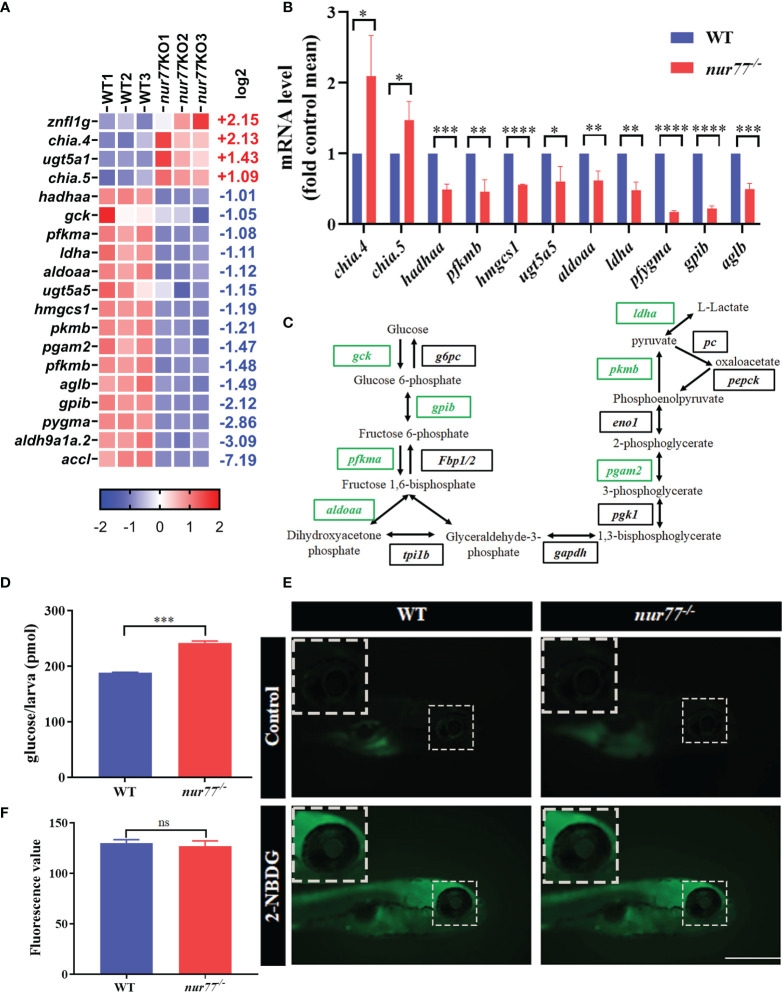
Nur77 regulates carbohydrate metabolism in zebrafish larvae. **(A)** Heatmaps of transcripts in carbohydrate metabolism enrichment. Colors represent high (red), low (blue), or average (white) expression values based on Z-score-normalized fragments per kilobase per million mapped read (FPKM) values for each gene. The Z-score indicators are shown under each map. The fold change (log2) is shown on the right. **(B)** The validation of the expression levels of differentially expressed genes by qRT-PCR analysis in the categories of carbohydrate metabolism (*n* = 3). **(C)** Disturbed glycolysis/gluconeogenesis. Blocks represent transcript-encoded enzymes. Green blocks represent downregulated genes, and black blocks represent unchanged genes. **(D)** The total free glucose contents of WT and *nur77* ^−/−^ zebrafish (*n* = 3). **(E)** 2-NBDG glucose uptake of wildtype and *nur77* ^−/−^ mutant larvae; the glucose uptake level is indicated by the fluorescence of lens (arrow) imaged by fluorescent microscopy. Wildtype and *nur77* ^−/−^ without 2-NBDG were used for control groups (upper panel). **(F)** Eye fluorescence intensity was measured based on images. Quantification of fluorescence intensity for WT and *nur77* ^−/−^ zebrafish larvae. Results were represented as means with standard errors (*n* = 3); ns, no significance; ^*^ *p* < 0.05, ^**^ *p* < 0.01, ^***^ *p* < 0.001, ^****^ *p* < 0.0001 by *t*-test.

There are 4 upregulated and 15 downregulated DEGs involved in 12 carbohydrate metabolic pathways (**Supplementary Table S5**; **Figure 5A**). Our qRT-PCR results of selected genes in carbohydrate metabolism were consistent with RNA-seq data (**Figure 5B**). Among the enriched pathways, the top 2 were glycolysis/gluconeogenesis (ko00010) (9 genes) and glucagon signaling pathway (ko04922) (6 genes), which are all involved in the glucose metabolism (**Supplementary Table S5**). In the glycolysis/gluconeogenesis pathway, the expression levels of all DEGs were decreased, including glucokinase (*gck*); pyruvate kinase, muscle b (*pkmb*); phosphofructokinase, muscle a/b (*pfkma/b*); glucose-6-phosphate isomerase b (*gpib*); lactate dehydrogenase A (*ldha*); phosphoglycerate mutase (*pgam2*); acetyl-CoA carboxylase-like (*accl*); aldehyde dehydrogenase 9 family, member A1a, tandem duplicate 2 (*aldh9a1a.2*); and acetyl-CoA carboxylase-like aldolase a, fructose-bisphosphate, a (*aldoaa*) (**Figure 5C**). *gck*, *pkmb*, and *pfkma/b* are the key enzymes in the glycolysis/gluconeogenesis pathway. *gck* is a member of the hexokinase family, which phosphorylates glucose to produce glucose-6-phosphate in pancreatic β cells and liver cells (57). *pfkma/b*, one isoform of phosphofructokinase, converts fructose-6-phosphate to fructose-1,6-bisphosphate in glycolytic pathway (58). *pkmb* catalyzes the conversion of phosphoenolpyruvate and ADP to pyruvate and ATP, which is the final step of glycolysis (59). Taken together, these data revealed that Nur77 deficiency in zebrafish led to the disorder of carbohydrate metabolism, especially in glucose metabolism.

To confirm the data from RNA-seq, we then measured the total free glucose in wildtype and *nur77^−/−^* larvae. As shown in **Figure 5D**, the glucose level in *nur77^−/−^* was significantly higher than wildtype. The increased free glucose may be due to disordered glycolysis/gluconeogenesis, impaired glucose uptake, and decreased insulin secretion. Since the signaling pathway analysis indicated that the glycolysis/gluconeogenesis pathway was impaired (**Figure 5C**), we then survey further the glucose uptake function in *nur77^−/−^* zebrafish and perform a 2-NBDG uptake assay according to Lee et al. (43). As indicated by the fluorescence intensity of lens, there was no significant increased glucose uptake in the *nur77^−/−^* group (**Figures 5E, F** **)**. These data suggested that higher-level free glucose in *nur77^−/−^* larvae was not due to glucose uptake but may be affected by the glycolysis/gluconeogenesis or β-cell function.

### Knockout of Nur77 Impaired β-Cell Function in Zebrafish

**Figure 6:**
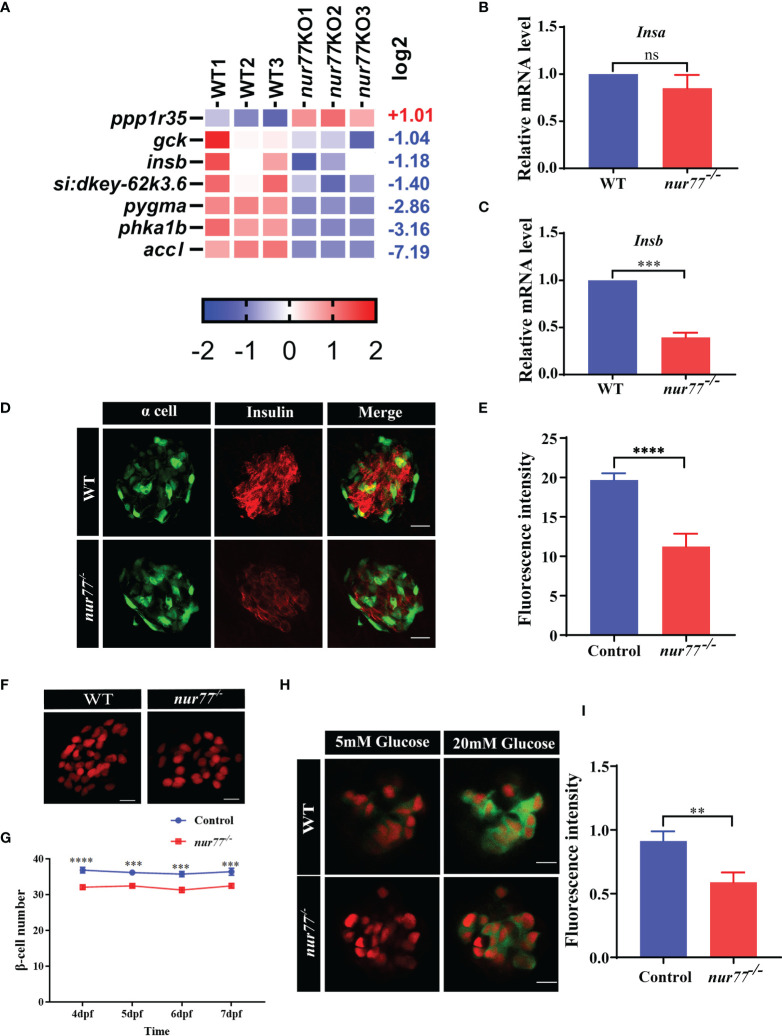
Nur77 regulates insulin secretion and β-cell number in zebrafish larvae. **(A)** Heatmaps of transcripts in insulin signaling pathway. Colors represent high (red), low (blue), or average (white) expression values based on Z-score-normalized fragments per kilobase per million mapped read (FPKM) values for each gene. **(B, C)** The validation of the expression levels of insulin genes by qRT-PCR analysis in WT and *nur77* ^−/−^ zebrafish larvae, **(B)** *insa* (*n* = 3), **(C)** and *insb* (*n* = 3). **(D, E)** The insulin contents in WT and *nur77* ^−/−^ zebrafish larvae. **(D)** Representative images of the fluorescence of α cell and β cell from control and *nur77* ^−/−^; α cells are indicated by the green fluorescence with *Tg(gcg:eGFP)*, β cell are indicated with red fluorescence by immunostaining with insulin antibody. **(E)** Quantification of β-cell fluorescence intensity from control and *nur77* ^−/−^. The number of larvae (*n* = 12~18). **(F, G)** The number of pancreatic β cells in *Tg(Ins:H2BmCherry)* and *nur77^−/−^;Tg(Ins:H2BmCherry)* zebrafish larvae. **(F)** Representative images of the β-cell (red) number in *Tg(Ins:H2BmCherry)* and *nur77^−/−^;Tg(Ins:H2BmCherry)* at 6 dpf. **(G)** Quantification of β-cell number in *Tg(Ins:H2BmCherry)* and *nur77^−/−^;Tg(Ins:H2BmCherry)* from 4 to 7 dpf zebrafish larvae (*n* = 19~50). **(H, I)** The glucose-stimulated GCaMP6s response in β cells of *Tg(Ins:H2BmCherry);Tg(Ins:GCaMP6s)* and *nur77^−/−^;Tg(Ins:H2BmCherry);Tg(Ins:GCaMP6s)*. **(H)** Representative images of GCaMP6s response in β cells of *Tg(Ins:H2BmCherry);Tg(Ins:GCaMP6s)* and *nur77^−/−^;Tg(Ins:H2BmCherry);Tg(Ins:GCaMP6s)* by 5 or 20 mM glucose ECS solution; green signal is GCaMP6. **(I)** Quantification of GCaMP6s response (GFP fluorescence intensity) in β cells of *Tg(Ins:H2BmCherry);Tg(Ins:GCaMP6s)* and *nur77^−/−^ nur77^−/−^;Tg(Ins:H2BmCherry);Tg(Ins:GCaMP6s)*. Results are represented as means with standard errors (*n* = 13~20); ns, no significance; ^**^ *p* < 0.01, ^***^ *p* < 0.001, ^****^ *p* < 0.0001 by *t*-test.

We then turned to analyze the DEGs in the insulin signaling pathway in the RNA-seq data. Interestingly, the *insb*, *gck*, and *accl* are significantly decreased (**Figure 6A**). We further confirmed that the expression level of *insb* but not *insa* decreased in *nur77^−/−^* larvae by qRT-PCR (**Figures 6B, C** **)**. Next, we evaluated the insulin content by immunofluorescence assay. As shown in **Figures 6D, E**, we found that the insulin content of *nur77^−/−^* larvae was significantly lower than that of wildtype larvae. We further investigated whether there were alterations with the β-cell mass in *nur77^−/−^* zebrafish. We crossed *nur77^−/−^* into β-cell reporter line, *Tg(Ins:H2BmCherry).* The number of β cells was significantly reduced in *nur77^−/−^; Tg(Ins:H2BmCherry)* (**Figures 6F, G** **)**. Moreover, to measure the insulin secretion, a live imaging of calcium influx reporter line, *Tg(Ins : GCaMP6s)* was applied. When insulin is secreted in response to glucose by pancreatic β cells, influx of calcium resulted in an increase of green fluorescence emission (42). Also, the intensity of fluorescence was proportional to the amount of insulin secretion. As shown in **Figures 6H, I**, when 6 dpf zebrafish embryos were treated with 5 mM glucose ECS solution, there is no obvious difference of green fluorescence in both *Tg(Ins:H2BmCherry); Tg(Ins:GCaMP6s)* and nur77^−/−^; *Tg(Ins:H2BmCherry); Tg(Ins:GCaMP6s)*. While after being stimulated by 20 mM glucose ECS solution, it produced a strong green signal in *Tg(Ins:H2BmCherry); Tg(Ins:GCaMP6s)*, but weak fluorescence in nur77^−/−^; *Tg(Ins:H2BmCherry); Tg(Ins:GCaMP6s) *(**Supplemental Videos 1, 2**). Taken together, these data suggested that knockout of *nur77* impaired β-cell function, including downregulated *insb* expression, reduced β-cell mass and suppressed insulin secretion.

### Nur77 Expressed Solely in β Cells Is Sufficient to Rescue the Defects in *Nur77* ^−/−^ Mutant

**Figure 7:**
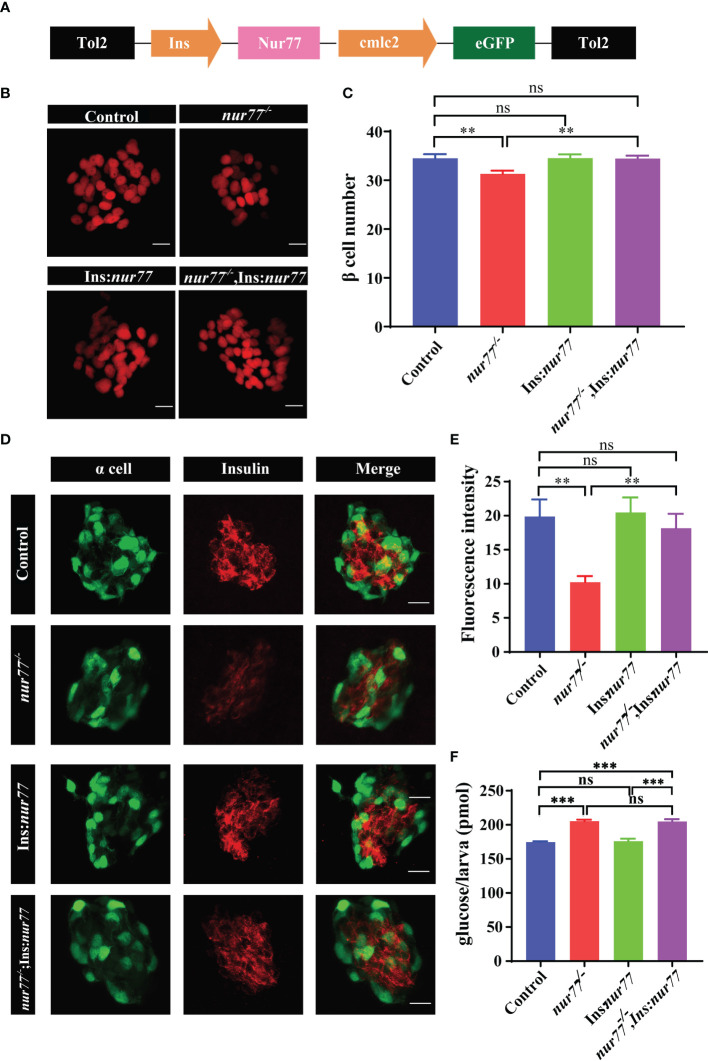
Targeted expression of Nur77 in β cells restored β-cell number and insulin content in zebrafish. **(A)** Schematic representation of the *Tg(Ins : Nur77; cmlc2:eGFP)* transgenic line and the expression pattern. Expression of *Nur77* was controlled by the zebrafish insulin promoter with *cmlc2*-driven eGFP used as an indication of transgene carriers. **(B, C)** Representative images **(B)** and quantification **(C)** of β-cell number in *Tg(Ins:H2BmCherry)*, *nur77^−/−^;Tg(Ins:H2BmCherry)*, *Tg(Ins : Nur77);Tg(Ins:H2BmCherry)* and *nur77^−/−^*; *Tg(Ins : Nur77)*;*Tg(Ins:H2BmCherry)* zebrafish larvae. The number of larvae (*n* = 20~30). **(D, E)** Representative images **(D)** and quantification **(E)** of the insulin fluorescence intensity in β cells from *Tg(gcg:eGFP), nur77^−/−^*;*(gcg:eGFP)*, *Tg(Ins : Nur77);Tg(gcg:eGFP)* and *nur77^−/−^*; *Tg(Ins : Nur77);Tg(gcg:eGFP)* zebrafish larvae. *Tg(gcg:eGFP)* was used to indicate the pancreatic α cell, and β cell are indicated with red fluorescence by immunostaining with insulin antibody (*n* = 12~15). **(F)** The total free glucose contents in *Tg(gcg:eGFP)*, *nur77^−/−^;(gcg:eGFP)*, *Tg(Ins : Nur77);Tg(gcg:eGFP)* and *nur77^−/−^*; *Tg(Ins : Nur77);Tg(gcg:eGFP)* zebrafish larvae (*n* = 3). ns, no significance; ^**^ *p* < 0.01, ^***^ *p* < 0.001 by one-way ANOVA.

We next asked whether Nur77 expression solely in the β cells is sufficient to rescue the β-cell defects in global *nur77^−/−^* mutants. We generated a transgenic line, *Tg(Ins:Nur77;cmlc2:eGFP)*, which targets expressed a human Nur77 cDNA under the control of the zebrafish insulin promoter solely in zebrafish β cells, and *cmlc2* promoter-driven eGFP used as an indication of transgene carriers (**Figure 7A**). Interestingly, when target expression of Nur77 in β cells under wildtype background, there was no change of β-cell number (control vs. *Tg(Ins:Nur77)*) (**Figures 7B, C** **)**. Whereas, in *nur77* mutant background, *nur77^−/−^;Tg(Ins:Nur77);Tg(Ins:H2BmCherry)* significantly increased β-cell number, which number is similar to the control group (**Figures 7B, C** **)**. Moreover, when we measured the insulin content by immunofluorescence assay, we found that *nur77^−/−^;Tg(Ins:Nur77)* larvae dramatically recovered their insulin fluorescence intensity (**Figures 7D, E** **)**. Furthermore, we also measured the free glucose level in *nur77^−/−^;Tg(Ins:Nur77)* zebrafish; their free glucose level also returned to the similarity of the control group (**Figure 7F**). These data suggested that the *nur77^−/−^-*impaired β-cell function was likely due to loss of Nur77.

## Discussion and Conclusion

Metabolic diseases such as obesity, nonalcoholic fatty liver disease, atherosclerosis, and diabetes are increasing worldwide and are becoming a global health concern. Hence, the development of new therapeutic approaches and novel drug targets are urgently needed. Accumulative evidence has proven that orphan nuclear receptor Nur77 is implicated in various metabolic processes, in particular, carbohydrate metabolism and lipid metabolism. Although Nur77 is an orphan nuclear receptor, several small molecules have been identified as regulators of Nur77. In terms of metabolism, CsnB from *Dothiorella* sp. HTF3 has been found to target Nur77 to increase blood glucose in fasting mice (25). Our previous studies found that celastrol, another Nur77 agonist, significantly reduced body weight, adipose tissue mass, and the size of adipocytes in HFD mice (32). Therefore, Nur77 may have great potential as a new drug target for metabolic diseases. To gain a global understanding of Nur77 and metabolism, as well as for screening small molecular drugs, we generated the *nur77* knockout zebrafish and applied RNA-seq analysis to reveal the metabolic regulatory networks of Nur77.

Although amino acid deficiency induces Nur77-related reticulophagy to maintain intracellular amino acid levels (60), there are no studies reporting about Nur77-regulated amino acid metabolism. It is the first time to give a global view of the altered genes of Nur77-related amino acid regulation. In our study, the transcription level of several key amino acid metabolism genes was significantly decreased (**Figure 3A**). These genes do not only perform their function in amino acid metabolism pathways but also in glucose and lipid metabolism. For instance, *hadhaa*, *hmgcs1*, *aldh9a1a.2*, and *aox5* (**Figure 3C**) are all in valine, leucine, and isoleucine degradation pathway; however, the downstream molecules of these pathway are crosstalked with fatty acid metabolism and tricarboxylic acid cycle (TCA cycle). Therefore, it is implicated that Nur77 participates in the crosstalk of the intracellular amino acid, lipid, and carbohydrate metabolic pathways.

Several studies have revealed the important roles of Nur77 signaling in regulation of lipid metabolism in mammals (16, 28, 61, 62). Similarly, we also observed that lipid metabolism was dysfunctional in *nur77* null larvae, showing the alteration of cholesterol, glycerolipid, steroid, and fatty acid metabolism pathways (**Supplementary Table S4**). Among DEGs, 5 of them involved in cholesterol biosynthesis, including *lss*, *cyp51*, *sqlea*, *sc5d*, and *hmgcs1* (**Figure 4C**). In addition, 4 DEGs in fatty acid metabolism were enriched, including *acc*, *ppt*, *hadhaa*, and *aldh* (**Supplementary Table S4**). Nur77 was reported to be involved in the modulation of β-oxidation facilitating melanoma cell survival under glucose deprivation (63). It is also reported that Nur77 regulates steroidogenic enzymes in ovarian theca cell in transcriptional level (64). These genes’ expression level changes in cholesterol biosynthesis and fatty acid metabolism may be associated with the elevated TC and TG content in *nur77* knockout zebrafish (**Figures 4D, E** **)**. In line with our data, whether in liver or adipose tissue, TC and TG will increase after high-fat diet in *Nur77* null mice (23, 65). Taken together, disruption of Nur77 function causes aberrant lipid metabolism.

19 DEGs were enriched in carbohydrate metabolism, and most of them are related to glucose homeostasis (**Figure 5A**). Indeed, the total free glucose was elevated in *nur77^−/−^* zebrafish mutant (**Figure 5D**). Normoglycemia is normally maintained through the central nervous system regulation of both hepatic glucose production and glucose uptake by muscle and adipose tissue (66–68). There are a variety of factors that contribute to elevated blood glucose, such as increased glucose uptake, decreased glycolysis, increased gluconeogenesis, insulin resistance, and insulin deficiency (69, 70). The pathway analysis indicated that glycolysis/gluconeogenesis pathway was impaired (**Figure 5C**). The genes in glycolysis, including *ldha*, *pkmb*, *pgam2*, and *gck*, are all reduced in the *nur77^−/−^* zebrafish mutant, suggesting that the glucose catabolism was decreased. However, two major rate-limiting enzymes that are involved in gluconeogenesis, *pepck* and *g6pc*, did not change in *nur77^−/−^* zebrafish (**Figure 5C**). These data suggested that decreased glycolysis may contribute to the elevated glucose. Moreover, we also examine the glucose uptake by using 2-NBDG uptake test, but there was no significant difference in wildtype and *nur77 ^−/−^* zebrafish (**Figures 5E, F** **)**.

We further demonstrated that the increased glucose in *nur77^−/−^* zebrafish was associated with the impaired β-cell function (**Figures 6**, **7**). In *nur77* knockout zebrafish larvae, the number of β cells was significantly reduced (**Figures 6F, G** **)**. Moreover, we revealed that knockout of Nur77 downregulated *insb* expression, decreased insulin content, and reduced insulin secretion in zebrafish β cells (**Figure 6**). Consistent with our results, studies reported that global knockout of *Nur77* results in a decrease in β-cell area in neonatal and young mice (71). Knockdown of Nur77 suppressed the β-cell proliferation in Ins-1 cell line and impedes production of ATP in mitochondria which lead to inhibit glucose-stimulated insulin secretion (14). Nur77 was suggested to protect against reactive oxygen species (ROS) or ER stress-induced β-cell apoptosis (31, 33). Whereas, a study reported that genetic deletion of *Nur77* did not exhibit any morphological differences in β cells compared with their WT littermates in mice (72). However, this conclusion was only based on insulin immunostaining (72).

In addition, we demonstrated that targeted expression of Nur77 in β cells of *nur77^−/−^* rescued the defects in β-cell function, as well as restored the normal glucose level (**Figure 7**). Although the detailed mechanism of Nur77 in the regulation of β-cell function needed to be further elucidated, studies have reported that Nur77 interplayed with many important β-cell transcription factors, such as mafA and Nkx6.1. It was reported that fatty acid stimulation increased Nur77 expression, Nur77 then interacted with FoxO1 to decrease intracellular concentration of the mafA protein, which prevents activation of the insulin gene, and resulted in a decreased intracellular insulin concentration and impaired insulin secretion (37). Overexpression of Nkx6.1 in rat pancreas induced β-cell proliferation through Nur77 and its homologous Nor1 (71). Target expression of Nur77 in zebrafish β cells restores the β-cell function probably due to Nur77 being re-enacted with these β-cell transcription factors.

In conclusion, we performed whole-organism RNA-seq of wildtype and *nur77^−/−^* mutant larvae. By bioinformatic analysis, we found that expression of many genes in metabolism-related pathways were altered more than twofold in *nur77^−/−^* compared with wildtype. These genes are mainly involved in amino acid, lipid, and carbohydrate metabolism. Further by experimental approaches, we revealed that *nur77^−/−^* mutant displays an increased TC and TG, alteration in total amino acids, and elevated glucose. Moreover, we demonstrated that the elevated glucose was not due to change of glucose uptake but was caused by the disorder of glycolysis/gluconeogenesis and impaired β-cell function, including downregulated *insb* expression, reduced β-cell mass, and suppressed insulin secretion. Furthermore, we also verified that targeted expression of Nur77 in the β cells is sufficient to rescue the β-cell defects in global *nur77^−/−^* mutants. These results provide new information about the global metabolic network that regulates Nur77 signaling, as well as the role of Nur77 in β-cell function. Consequently, the *nur77* knockout zebrafish model will be a systematic, economic, effective animal model for further mechanism investigation of Nur77-associated metabolism regulation and screening Nur77-related small molecule compounds.

## Data Availability Statement

The original contributions presented in the study are publicly available. This data can be found here: NCBI, BioProject, PRJNA801348.

## Ethics Statement

The animal study was reviewed and approved by the Animal Care and Utilization Committee of Xiamen University (Protocol No. XMULAC20160089 of March 10, 2016). Written informed consent was obtained from the owners for the participation of their animals in this study.

## Author Contributions

ML and JL are the guarantors of this work and, as such, had full access to all of the data in the study and take responsibility for the integrity of the data and the accuracy of the data analysis. ML, JL, and YX designed the study. JT, YX, QK, HY, CL, and ZL performed key experiments. ML, JL, YX, QK, HY, CL, and ZL participated in the planning of the work and the interpretation of the results. YX drafted the manuscript. JL and ML revised the paper. All authors listed have made a substantial, direct, and intellectual contribution to the work and approved it for publication.

## Funding

This work was supported by the Natural Science Foundation of Fujian Province of China (grant numbers 2020J01042 to JL, 2019J01036 to YX). This work was supported by grants from the Natural Science Foundation of China (81670709 to ML), Key Laboratory of Marine Biotechnology of Fujian Province (2021MB02 to ML), and by by Grant 3502Z20184027 from the Natural Science Foundation of Xiamen.

## Conflict of Interest

The authors declare that the research was conducted in the absence of any commercial or financial relationships that could be construed as a potential conflict of interest.

## Publisher’s Note

All claims expressed in this article are solely those of the authors and do not necessarily represent those of their affiliated organizations, or those of the publisher, the editors and the reviewers. Any product that may be evaluated in this article, or claim that may be made by its manufacturer, is not guaranteed or endorsed by the publisher.

## Abbreviations

DEGs, differentially expressed genes; GO, Gene Ontology; KEGG, Kyoto Encyclopedia of Gene and Genomes; HFD, high-fat diet; ER, endoplasmic reticulum; RNA-Seq, RNA sequencing; PCR, polymerase chain reaction; Ds cDNA, double-stranded cDNA; qRT-PCR, real-time quantitative reverse transcription PCR; 2-NBDG, 2-[N-(7-nitrobenz-2-oxa-1,3-diazol-4-yl) amino]-2-deoxy-d-glucose; FBS, fetal bovine serum; Dox, doxycycline; dpf, days postfertilization; DMSO, dimethyl sulfoxide; eGFP, enhanced green fluorescent protein; ECS, extracellular solution; PCA, principal component analysis; NAD, nicotinamide adenine dinucleotide; UGTs, UDP-glucuronosyl transferases; TC, total cholesterol; TG, triglyceride; ATP, adenosine triphosphate; ADP, adenosine diphosphate; WT, wild type; TCA cycle, tricarboxylic acid cycle; CsnB, cytosporone B; ROS, reactive oxygen species; SREBP1c, sterol regulatory element-binding protein 1c; *hadhaa*, hydroxyacyl-CoA dehydrogenase trifunctional multienzyme complex subunit alpha a; *hmgcs1*, 3-hydroxy-3-methylglutaryl-CoA synthase 1; *aldh9a1a.2*, aldehyde dehydrogenase family 9 member A1; *aox5*, aldehyde oxidase; *sqlea*, squalene epoxidase a; *lipca*, lipase hepatic a; *msmol*, methylsterol monooxygenase 1; *cyp51*, Sterol 14α-demethylase; *gck*, glucokinase; *pkmb*, pyruvate kinase, muscle b; *pfkma/b*, phosphofructokinase, muscle a/b; *gpib*, glucose-6-phosphate isomerase b; *ldha*, lactate dehydrogenase A; *pgam2*, phosphoglycerate mutase; *accl*, acetyl-CoA carboxylase-like; *aldoaa*, acetyl-CoA carboxylase-like aldolase a, fructose-bisphosphate, a.
