# Supplementary material for: Knockout of Nur77 Leads to Amino Acid, Lipid, and Glucose Metabolism Disorders in Zebrafish
Source: Front Endocrinol (Lausanne). 2022 Apr 25;13:864631. doi: 10.3389/fendo.2022.864631 (PMC9084189; doi:10.3389/fendo.2022.864631)
Supplement: Supplementary file 1 [file DataSheet_1.zip › Supplemental materials 20220330/Supplemental Figure 1 and Figure 2.docx]

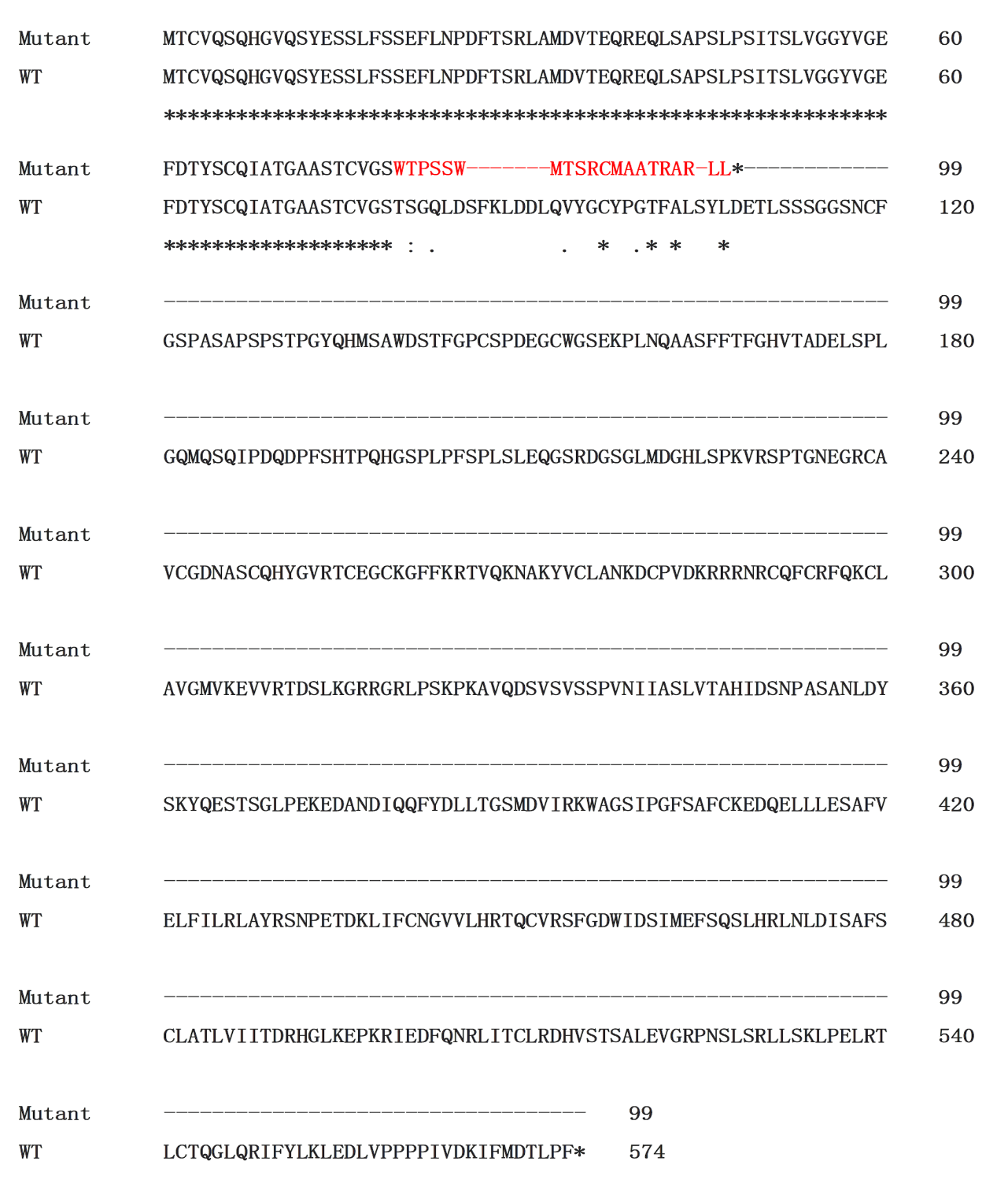


Supplemental figure 1. Sequence comparison of WT Nur77 and 13 bp deletion mutation. Sequence alignments were obtained with the Clustal method.


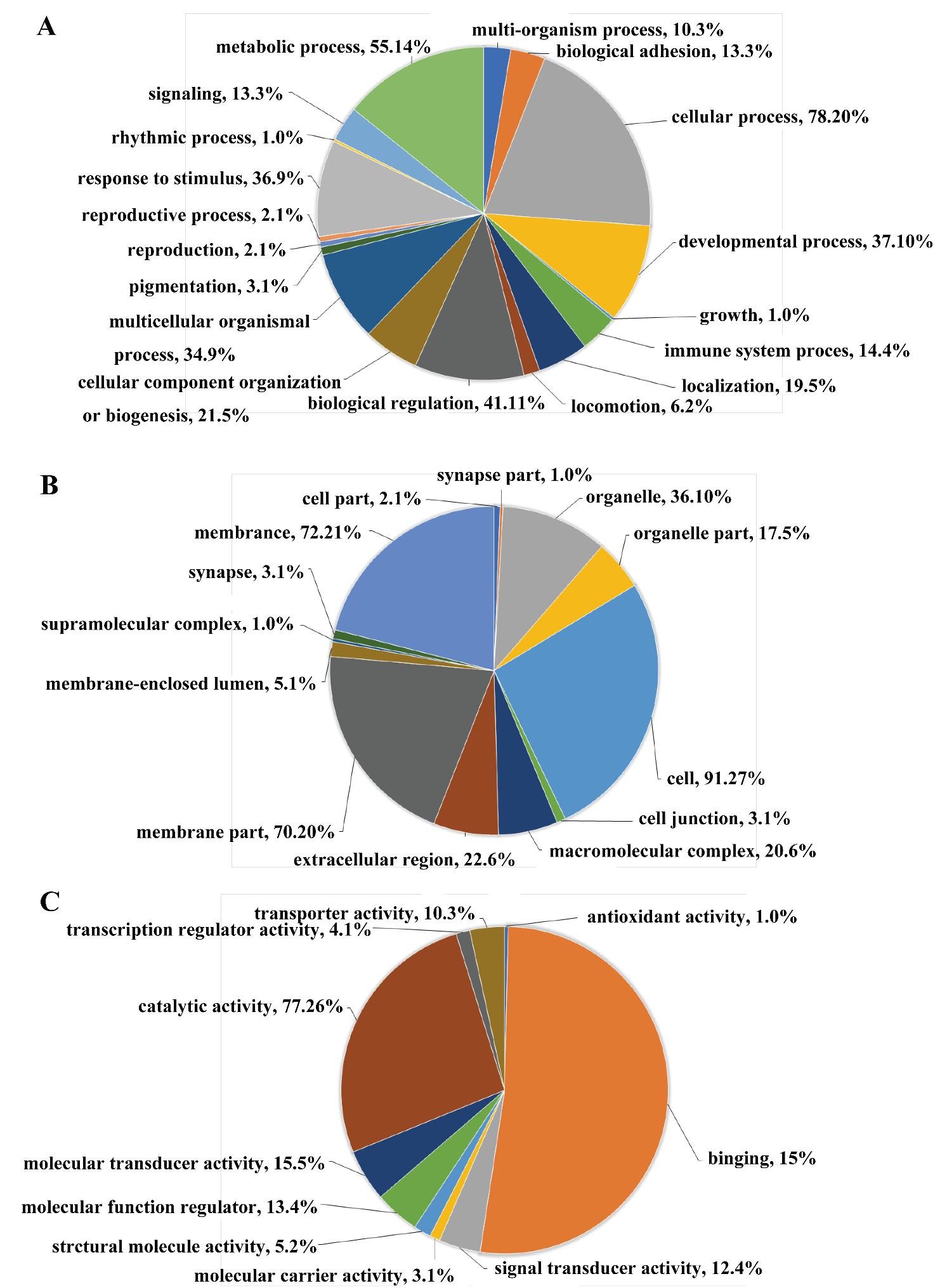


**Supplemental figure 2** A-C. Gene ontology (GO) enrichment analysis of DEGs in *nur77^-/-^* mutant zebrafish. The DEGs were classified into three categories: biological process (A), cellular component (B) and molecular function (C). The names of the GO subcategories and the percentage of each subcategory are showed around the three pie charts.
