# Supplementary material for: Knockout of Nur77 Leads to Amino Acid, Lipid, and Glucose Metabolism Disorders in Zebrafish
Source: Front Endocrinol (Lausanne). 2022 Apr 25;13:864631. doi: 10.3389/fendo.2022.864631 (PMC9084189; doi:10.3389/fendo.2022.864631)
Supplement: Supplementary file 1 [file DataSheet_1.zip › Supplemental materials 20220330/Supplemental Table. 3 Amino acid metabolism genes.docx]

**Amino Acid metabolism genes**

| **KEGG Canonical Pathways** | **Ko No** | **No of genes** | **Gene symbols**  **(log2 fold change)** |
| --- | --- | --- | --- |
| Alanine, aspartate and glutamate metabolism | ko00250 | 1 | *adssl1*(-1.33) |
| Glycine, serine and threonine metabolism | ko00260 | 2 | *cthl*(1.38); *pgam2*(-1.47) |
| Cysteine and methionine metabolism | ko00270 | 2 | *ldha*(-1.11); *cthl*(1.38) |
| Valine, leucine and isoleucine degradation | ko00280 | 4 | *hmgcs1*(-1.19) *hadhaa*(1.01); *aldh9a1a.2*(-3.09); *aox5*(-1.27); |
| Lysine degradation | ko00310 | 3 | *hadhaa*(-1.01); *aldh9a1a.2*(-3.09); *setd5l* (-3.77) |
| Arginine and proline metabolism | ko00330 | 2 | *aldh9a1a.2*(-3.09); *odc1*(-1.13) |
| Histidine metabolism | ko00340 | 1 | *aldh9a1a.2*(-3.09); |
| Tyrosine metabolism | ko00350 | 1 | *aox5*(-1.27) |
| Tryptophan metabolism | ko00380 | 3 | *hadhaa*(-1.01); *aldh9a1a.2*(-3.09); *aox5*(-1.27) |
| Biosynthesis of amino acids | ko01230 | 2 | *cthl*(1.38); *pgam2*(-1.47) |
