# Supplementary material for: Knockout of Nur77 Leads to Amino Acid, Lipid, and Glucose Metabolism Disorders in Zebrafish
Source: Front Endocrinol (Lausanne). 2022 Apr 25;13:864631. doi: 10.3389/fendo.2022.864631 (PMC9084189; doi:10.3389/fendo.2022.864631)
Supplement: Supplementary file 1 [file DataSheet_1.zip › Supplemental materials 20220330/Supplemental Table. 4 Lipid metabolism genes.docx]

**Lipid metabolism genes**

| **KEGG Canonical Pathways** | **Ko No** | **No of genes** | **Gene symbols**  **(log2 fold change)** |
| --- | --- | --- | --- |
| Fatty acid biosynthesis | ko00061 | 1 | *accl*(-7.19) |
| Fatty acid elongation | ko00062 | 2 | *ppt2a.1*(-2.35); *hadhaa*(-1.01) |
| Fatty acid metabolism | ko01212 | 3 | *ppt2a.1*(-2.35); *hadhaa*(-1.01); *accl*(-7.19) |
| Fatty acid degradation | ko00071 | 2 | *aldh9a1a.2*(-3.09); *hadhaa*(-1.01) |
| Steroid biosynthesis | ko00100 | 5 | *msmo1*(-1.13); *cyp51*(-1.15); *sc5d*(-1.17); *lss*(-1.64); *sqlea*(-1.42) |
| Steroid hormone biosynthesis | ko00140 | 2 | ugt5a5(-1.15); ugt5a1(1.43) |
| Glycerolipid metabolism | ko00561 | 4 | *slc9b2l* (1.21); *pnpla1*(-1.12); *lipca*(-1.76); *aldh9a1a.2*(-3.09) |
| Arachidonic acid metabolism | ko00590 | 2 | *selenou1a* (-6.41); *cyp2p9*(1.29) |
| Linoleic acid metabolism | ko00591 | 1 | *cyp2p9*(1.29) |
| Cholesterol metabolism | ko04979 | 1 | *lipca*(-1.76) |
| MAPK pathway | ko04010 | 1 | *selenou1a* (-6.41) |
