# Supplementary material for: Knockout of Nur77 Leads to Amino Acid, Lipid, and Glucose Metabolism Disorders in Zebrafish
Source: Front Endocrinol (Lausanne). 2022 Apr 25;13:864631. doi: 10.3389/fendo.2022.864631 (PMC9084189; doi:10.3389/fendo.2022.864631)
Supplement: Supplementary file 1 [file DataSheet_1.zip › Supplemental materials 20220330/Supplemental Table. 5 Carbonhydrate metabolism genes.docx]

**Carbohydrate metabolism genes**

| **KEGG Canonical Pathways** | **Ko No** | **No of genes** | **Gene symbols (log2 fold change)** |
| --- | --- | --- | --- |
| Glycolysis / Gluconeogenesis | ko00010 | 9 | *aldh9a1a.2*(-3.09); *gpib*(-2.12); *ldha*(-1.11); *aldoaa*(-1.12); *pkmb*(-1.21); *pfkma*(-1.08); *pfkmb*(-1.48); *pgam2*(-1.47); *gck*(-1.04) |
| Pentose and glucuronate interconversions | ko00040 | 2 | *ugt5a5*(-1.15); *ugt5a1* (1.43) |
| Fructose and mannose metabolism | ko00051 | 4 | *aldoaa*(-1.12); *pfkma*(-1.08); *pfkmb*(-1.48); *znfl1g* (2.15) |
| Galactose metabolism | ko00052 | 3 | *pfkma*(-1.08); *pfkmb*(-1.48); *gck*(-1.04) |
| Ascorbate and aldarate metabolism | ko00053 | 3 | *aldh9a1a.2*(-3.09); *ugt5a5*(-1.15); *ugt5a1* (1.43) |
| Starch and sucrose metabolism | ko00500 | 4 | *gpib*(-2.12); *aglb*(-1.49); *pygma*(-2.86); *gck*(-1.04) |
| Amino sugar and nucleotide sugar metabolism | ko00520 | 4 | *gpib*(-2.12); *chia.4*(2.13); *chia.5*(1.09); *gck*(-1.04) |
| Pyruvate metabolism | ko00620 | 4 | *aldh9a1a.2*(-3.09);*Accl*(-7.19); *ldha*(-1.11); *pkmb*(-1.21) |
| Propanoate metabolism | ko00640 | 3 | *accl*(-7.19); *ldha*(-1.11); *hadhaa*(-1.01) |
| Butanoate metabolism | ko00650 | 2 | *hmgcs1*(-1.19); *hadhaa*(-1.01) |
| Glucagon signaling pathway | ko04922 | 6 | *accl*(-7.19); *ldha*(-1.11); *pkmb*(-1.21); *pygma*(-2.86); *gck*(-1.04); *pgam2*(-1.47) |
| Insulin resistance | ko04931 | 1 | *pygma*(-2.86) |
